# Complete Genome Sequences of the Human Pathogen Paenibacillus thiaminolyticus Mbale and Type Strain P. thiaminolyticus NRRL B-4156

**DOI:** 10.1128/MRA.00181-20

**Published:** 2020-04-09

**Authors:** Christine Hehnly, Lijun Zhang, Joseph N. Paulson, Mathieu Almeida, Benjamin von Bredow, Dona S. S. Wijetunge, Michael Y. Galperin, Kathryn Sheldon, Steven J. Schiff, James R. Broach

**Affiliations:** a Penn State Institute for Personalized Medicine, The Pennsylvania State University College of Medicine, Hershey, Pennsylvania, USA; b Department of Biochemistry and Molecular Biology, The Pennsylvania State University College of Medicine, Hershey, Pennsylvania, USA; c Department of Biostatistics, Product Development, Genentech, Inc., South San Francisco, California, USA; d MetaGénoPolis, INRA, Université Paris-Saclay, Jouy-en-Josas, France; e Department of Pathology, The Pennsylvania State University College of Medicine, Hershey, Pennsylvania, USA; f National Center for Biotechnology Information, National Library of Medicine, National Institutes of Health, Bethesda, Maryland, USA; g Department of Neurosurgery, The Pennsylvania State University College of Medicine, Hershey, Pennsylvania, USA; h Penn State Center for Neural Engineering, The Pennsylvania State University College of Engineering, University Park, Pennsylvania, USA; i Department of Engineering Science and Mechanics, The Pennsylvania State University College of Engineering, University Park, Pennsylvania, USA; j Department of Physics, The Pennsylvania State University Eberly College of Science, University Park, Pennsylvania, USA; Queens College

## Abstract

We have isolated a likely bacterial pathogen from cerebrospinal fluid from a Ugandan infant suffering from hydrocephalus. Whole-genome sequencing and assembly of the genome of the clinical isolate, as well as that of a previously deposited reference strain, identified the isolate as Paenibacillus thiaminolyticus, which has not been associated with widespread human infections.

## ANNOUNCEMENT

The *Paenibacillus* genus has been studied widely for its industrial and agricultural associations (1) and as the pathogen causing American foulbrood disease among honey bee larvae (2). Although they cause occasional opportunistic infections in humans (3), *Paenibacillus* species have not been associated with widespread human infections. However, our recent report associated a *Paenibacillus* sp. with postinfectious hydrocephalus in infants in sub-Saharan Africa (J. N. Paulson, B. L. Williams, C. Hehnly, N. Mishra, S. Sinnar, L. Zhang, P. Ssentongo, E. Mbabazi-Kabachelor, D. S. S. Wijetunge, B. von Bredow, R. Mulondo, J. Kiwanuka, F. Bajunirwe, J. Bazira, L. Bebell, K. Burgoine, M. Couto-Rodriguez, J. E. Ericson, T. Erickson, M. Ferrari, M. Gladstone, C. Guo, M. Haran, M. Hornig, A. M. Isaacs, B. N. Kaaya, A. V. KulKarni, E. Kumbakumba, X. Li, D. D. Limbrick, Jr, J. Magombe, S. U. Morton, J. Mugamba, J. Ng, P. Olupot-Olupot, J. Onen, F. Roy, K. Sheldon, R. Townsend, A. D. Weeks, A. J. Whalen, J. Quackenbush, P. Ssenyonga, M. Y. Galperin, M. Alemeida, H. Atkins, B. C. Warf, W. I. Lipkin, J. Broach, and S. J. Schiff, unpublished data). Presented here is the clinical isolate Paenibacillus thiaminolyticus Mbale, which was recovered from a patient in Mbale, Uganda. We assembled a draft genome using optical mapping and error-corrected long-read sequencing of the clinical isolate and the type strain P. thiaminolyticus NRRL B-4156, which was isolated in 1950 from a thiamine-deficient patient (4).

Paenibacillus thiaminolyticus was recovered from previously fresh-frozen cerebrospinal fluid, from an infant in Uganda with postinfectious hydrocephalus, by growth in a lytic anaerobic blood culture bottle (Bactec, BD) at 37°C for 14 days. High-molecular-weight DNA was prepared according to the blood and cell culture DNA isolation protocol with lysozyme digestion (Bionano Genomics, CA, USA).

A PCR-free short-read sequencing library for the clinical isolate was prepared from 1 μg of DNA fragmented with an E220 focused ultrasonicator (Covaris), with an average insert size of 400 bp, using a HyperPrep kit (KAPA Biosystems). The final library (10 pM) was sequenced to 20 million reads using MiSeq 600-cycle v3 reagents (Illumina). Previously reported short-read sequencing data were used for the type strain NRRL B-4156 (5).

Long-read sequencing libraries for the type strain and the clinical isolate were prepared using a HyperPrep kit and 1D sequencing kit and were sequenced on the MinION platform with an SQK-LSK108 SpotON flow cell (Oxford Nanopore Technologies, UK). Base calling and trimming using Albacore v2.3.1 (https://github.com/dvera/albacore) and Porechop v0.2.3 (https://github.com/rrwick/Porechop) resulted in 130,472 and 559,846 reads for the type strain and clinical isolate, respectively. We assembled long-read genomes using Canu v1.7 (6) and then corrected them with short-read sequences using Pilon v1.22 (7) and Bowtie 2 v2.3.4.3 (8). All software programs were run using default settings. We obtained 6 contigs, with an *N*_50_ of 2.26 Mbp, for the clinical isolate and 1 continuous 6,612,442-bp contig for the type strain. The type strain assembly is likely the entire chromosome.

Optical mapping of the clinical isolate DNA on a Bionano Genomics Saphyr system, following direct labeling and staining protocol revision A (Bionano Genomics), resulted in a continuous 7.6-Mbp linear chromosome with overlapping ends, with 70× effective coverage. Three fragmented contigs from the corrected long-read assembly were aligned to the optical map, yielding a 6,744,575-bp hybrid scaffold, using Bionano Access v1.3.0, Bionano Tools v1.3.8041.8044, and Hybrid Scaffold v10252018.

Three phage insert regions were found in the type strain genome and 11 phage regions were found in the clinical isolate genome using PHASTER (9). Two of the 11 regions were identified on 99,275-bp and 35,613-bp contigs, which identified them as likely extrachromosomal phages of unknown origin. The remaining 52,271-bp contig had 37,930 bp that mapped back to the hybrid scaffold and another 14,393 bp, both with 99.8% similarity. From optical mapping, a 40-kbp insertion lies at position 4098996 in the hybrid scaffold, which correlates with the remaining contig. Further investigations would verify whether this is a technical or biological event.

The 16S rRNA genes of the two genomes had 99.2 to 99.4% sequence similarity, with a genomic average nucleotide identity (ANI) of 97.06%, as determined by the ANI calculator (10). Phylogenetic analysis placed the clinical isolate between two P. thiaminolyticus species, using 40 conserved markers that were extracted and aligned from the annotated genome (11) using fetchMG v1.0 (12) and MUSCLE v3.8.425 (13).

### Data availability.

The whole-genome assemblies, shotgun short reads, and MinION reads were deposited in GenBank (accession numbers PRJNA552221, PRJNA552222, SRR11015844, and SRR11031176).
